# Topical application of lidocaine or bupivacaine in the healing of surgical wounds in dogs^1^

**DOI:** 10.1590/s0102-865020200070000001

**Published:** 2020-08-14

**Authors:** Bruno Watanabe Minto, Laura Zanato, Guilherme Galhardo Franco, Fernando Yoiti Kitamura Kawamoto, Camila Potério Borsaro, Josiane Morais Pazzini, Elizabeth Regina Carvalho, Andresa Matsui

**Affiliations:** I Full Professor, Department of Clinical and Veterinary Surgery, Faculdade de Ciências Agrárias e Veterinárias, Universidade Estadual Paulista (FCAV-UNESP), Jaboticabal-SP, Brazil. Conception and design of the study, technical procedures, manuscript preparation and writing, critical revision, final approval.; II Graduate student, FCAV-UNESP, Jaboticabal-SP, Brazil. Acquisition, analysis and interpretation of data; manuscript preparation.; III Post-doctorate, Postgraduate Program in Veterinary Surgery, FCAV-UNESP, Jaboticabal-SP, Brazil. Conception and design of the study, analysis and interpretation of data, technical procedures, statistics analysis, manuscript preparation and writing, critical revision, final approval.; IV Fellow PhD degree, Postgraduate Program in Veterinary Surgery, FCAV-UNESP, Jaboticabal-SP, Brazil. Acquisition of data, technical procedures, statistics analysis, manuscript preparation and writing, critical revision, final approval.; V Full Professor, União das Faculdades dos Grandes Lagos (UNILAGO), Sao Jose do Rio Preto-SP, Brazil. Manuscript preparation and writing.; VI Fellow PhD degree, Postgraduate Program in Veterinary Surgery, FCAV-UNESP, Jaboticabal-SP, Brazil. Acquisition, analysis and interpretation of data; technical procedures; manuscript preparation.; VII Fellow PhD degree, Postgraduate Program in Veterinary Surgery, FCAV-UNESP, Jaboticabal-SP, Brazil. Conception and design of the study; histopathological examinations; acquisition, analysis and interpretation of data; manuscript preparation.

**Keywords:** Surgical Wounds, Lidocaine, Bupivacaine, Analgesia, Dogs

## Abstract

**Purpose:**

To analyze the anesthetic drugs interference with wound healing when used in the surgical bed.

**Methods:**

Macro and microscopic aspects of healing of surgical wounds were evaluated after instillation of topical anesthetics without vasoconstrictor or saline solution 0.9% as control in the transsurgical period. Thirty dogs, males and females were divided into two experimental groups. In both groups, two circular punch lesions of 6 mm diameter were performed in the abdomen. In group 1, lidocaine was instilled in one of the lesions and saline solution in the contralateral lesion. In group 2 the procedure was repeated with the use of bupivacaine. The macroscopic assessment of the lesions was performed on the first, third and tenth postoperative day. The excisional biopsy was performed on the tenth day and the samples were submitted for histopathological examination.

**Results:**

The macroscopic analysis had a significant difference between groups. Microscopic analysis was not significant between groups.

**Conclusions:**

The topical application of lidocaine and bupivacaine in the surgical wound is feasible and it does not influence skin healing. The benefit of such a practice, which has been the subject of other studies, seems to outweigh the risks.

## Introduction

In the face of scientific, clinical and economic interests, wound healing is the subject of several studies and current research^1^ ^,^ ^2^.

Normal healing is a complex and multifaceted process that involves continuous exchange of information between cells and the extracellular matrix^1^. This exchange of information occurs through the interaction between cells and between them and the matrix. This process occurs through direct contact or through chemical compounds. Most of the compounds involved in communication in the healing process are cytokines, soluble proteins secreted by cells and growth factors^2^ ^,^ ^3^.

The quality of healing is influenced by several factors. The type, size, depth and location of the wound, apposition of the edges, contamination, vascular supply, movement and metabolic and nutritional conditions of the patient, exemplify the main ones^3^.

Substances are used in surgical wounds to accelerate healing, prevent or treat infections, decrease pain or remove dead tissue^4^. The instillation of local anesthetics in surgical wounds is one of the techniques used to reduce intraoperative nociception or postoperative^5^ ^–^ ^7^ pain. It can be part of what is known as multimodal analgesia, which consists of the use of combined anti-pain techniques^6^ ^,^ ^7^. Among the anesthetic drugs most used for this practice are amides, especially lidocaine and bupivacaine^8^ ^,^ ^9^. In general, these drugs can be administered with minimal tissue irritation and unlikely to induce allergic reactions or systemic toxicity^9^. However, the effects of local anesthesia on healing are not well known^10^ ^,^ ^11^.

The use of local anesthetics instilled (splashblock) in surgical wounds is considered by some authors as a simple, safe and effective procedure in pain management, and can be used as one of the techniques in multimodal analgesia^12^.

However, others point out that, in addition to the ideal and technical doses not being well defined, there are reports of deleterious effects on wound healing^12^. Few scientific studies have been carried out to determine whether the effects of these drugs on healing are beneficial, harmful or non-existent. This fact encouraged and justifies the performance of the present study.

## Methods

This study was approved by the Committee on Ethics in the Use of Animals (CEUA), Universidade Estadual Paulista (UNESP), campus Jaboticabal (Protocol number 22733/2016).

### Experimental animals and groups

The procedures of this study were performed at the “Governador Laudo Natel” Veterinary Hospital of the Faculty of Agrarian and Veterinary Sciences (FCAV), UNESP, campus Jaboticabal.

Patients were recruited from the service routine of the Teaching Hospital of the Faculty of Agricultural Sciences -FCAV / UNESP, and the Zoonosis Control Center and Animal Protection Association (APA) site. All animal tutors agreed with the experiment, according to the project's methodology.

Thirty animals were used, randomly divided into two experimental groups, of varying characteristics, breeds and sizes. The minimum age was six months and the maximum was six years, with the majority being between one and two years old. The characteristics of the coat varied in length, color and type. The lightest animal weighed 4,7kg and the largest 22,4kg. Almost all the animals had not breed, except for a Border Collie and a Shih-tzu. Twenty females and ten males were used, and the genders were divided equally in each group.

### Anesthetic procedures

All patients were anesthetized with pre-anesthetic medication (MPA), which consisted of intramuscular morphine (0.5mg/kg). The animals were catheterized and received intravenous meloxicam (0.2mg/kg) and subcutaneous enrofloxacin (5mg/kg). Anesthetic induction was performed with intravenous propofol (5mg / kg). Subsequently, after intubation, anesthesia was maintained by combination of oxygen with inhaled isoflurane anesthesia, minimal concentration sufficient to maintain the patient on adequate anesthesia. In the intraoperative period, fentanyl (2.5µg/kg) was used, when necessary. After anesthetic recovery, the animals were accommodated in individual stalls and received Elizabethan collars. Water and food were offered ad libitum.

### Surgical procedure

The dogs were submitted to ovariosalpingohisterectomy (20 animals) or orchiectomy (10 animals).

Simultaneously with the surgical procedure, cutaneous surgical wounds were created with the aid of a disposable 0.6mm punch, at the height of the umbilical scar, and at the same distance from it on both sides of the abdomen. In the lesion of the right antimere, 0.1mL of lidocaine (20mg/mL) was instilled in 15 animals (Group 1) and 0.1mL of bupivacaine (5mg/mL) in another 15 (Group 2). In the lesion of the left antimere, in turn, 0.1mL of sterile 0.9% saline solution was instilled in all animals. After approximately one minute, the surgical wounds were sutured with 3-0 monofilament nylon thread, with two separate stitches. At the end of the surgical procedure, the wounds were cleaned with gauze soaked in sterile 0.9% saline solution, protected with gauze pad and tape Micropore^®^.

### Healing period

The animals were accommodated in individual cages, with hollow floorboards and water and food ration *ad libitum*, and the ration offered was what they were used to.

The dogs were supervised daily, at least twice a day, in order to observe their feces, urine and their food level. During this period, they had access to the solarium and time to socialize, if they had already lived together, and to receive affection and attention. The animals received 0.1mg / kg of meloxicam and 5mg / kg of enrofloxacin subcutaneously for two days.

### Analysis

Surgical wounds were analyzed daily for dehiscence, infection or any complication that could affect the animals’ health. Wounds performed by the punch were evaluated macroscopically on days 1, 3 and 10 to monitor the evolution of healing in terms of edema, erythema, pain or discomfort, vocalization to manipulation, pruritus, secretion and general healing evolution (Table 1).

**Table 1:** Surgical procedure in dogs. Scoring system for macroscopic evaluation of surgical wound.

Macroscopic Variables	0	1	2	3	4
Edema	Absent	Light	Moderate	Intense	-
Erythema	Absent	Light	Moderate	Intense	-
Pain	Absent	Light	Moderate	Intense	-
Vocalization	Absent	Light	Moderate	Intense	-
Prurience	Absent	Light	Moderate	Intense	-
Secretion	Absent	Serous	Ceruminous	Purulent	Mucoid
Amount of secretion	Absent	Light	Moderate	Intense	-
Cicatrization	-	Good	Regular	Bad	-

It was performed excisional biopsy scars formed on both lesions created by the punch on the tenth day after surgery. The same aseptic and anesthetic techniques previously described for surgery were used. The healing area was excised with a 0.6 mm disposable punch and packed in 10% buffered formaldehyde.

After 10% formalin fixation, all samples were embedded in paraffin and cut into 5-micrometer sections. Two slides of each sample were made, performing histochemical stains of hematoxylin-eosin and Masson's trichrome to evaluate collagenization. Histopathological analysis was performed following a scoring system (0 to 3), in which collagenization, neovascularization, edema, degree of acute and chronic inflammatory cells were evaluated.

Histopathological analysis was performed at the Department of Veterinary Pathology at UNESP/FCAV, Jaboticabal-SP. The analyzed items are described in Table 2.

**Table 2:** Surgical procedure in dogs. Scoring system for microscopic evaluation of surgical wound.

Microscopic Variables	0	1	2	3
Collagenization	Absent	Light	Moderate	Intense
Neovascularization	Absent	Light	Moderate	Intense
Edema	Absent	Light	Moderate	Intense
Acute inflammatory cells	Absent	Light	Moderate	Intense
Chronic inflammatory cells	Absent	Light	Moderate	Intense
Collagenization	Absent	Light	Moderate	Intense
Neovascularization	Absent	Light	Moderate	Intense
Edema	Absent	Light	Moderate	Intense

### Statistical analysis

For comparison between groups (1 and 2) and injuries (A and B) in relation to categorical variables (presence and degree of edema, presence and degree of erythema, presence and degree of pain / discomfort, vocalization to manipulation, presence and degree of pruritus, presence and characterization of secretion, amount of secretion, evolution of the healing process, collagenization, vascularization, edema and degree of acute and chronic inflammatory cells) the Kruskal-Wallis test was used, with subsequent use of the comparison test Dunn's multiple, for cases in which there was a significant difference between the medians of the groups. For all tests, p values equal to or less than 0.05 (p <0.05) were considered significant. For the analyses, we used the npar1way procedure, from the SAS computer programs (SAS 9.1, SAS Institute, Cary. NC, USA) and the GraphPadPrism program, Version 4.00.

## Results

**Figure 1:**
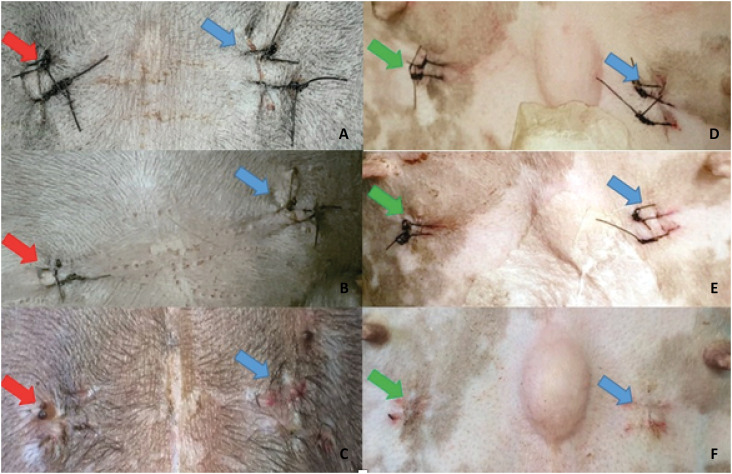
Photographic images of the macroscopic aspect of surgical wounds. (**A**), (**B**) and (**C**): treatment with bupivacaine (*red arrow*) and control (*blue arrow*) after 1, 3 and 10 days after surgery, respectively. (**D**), (**E**) and (**F**): treatment with lidocaine (*green arrow*) and control (*blue arrow*) after 1, 3 and 10 days after surgery, respectively.

All patients presented closure of the surgical wound on the tenth day, when the stitches were removed and the excisional biopsy (Fig. 1). There were no cases of infection or any clinical sign of complication in the period in which they were observed.

The period were uneventful dehiscence points with maintenance of tissue integrity in two patients, one from each group. In both cases, the suture loss occurred in the right antimere and the wound was treated with bupivacaine. In one of them the healing occurred by second intention, while in the other the wound had already healed.

One patient had discreet pruritus in the left antimere and moderate pruritus in the right antimere, on which side bupivacaine was applied. Another showed mild pruritus in both antimers.

The variables observed presence and degree of pain/discomfort, vocalization to manipulation, presence and characterization of secretion and amount of secretion were absent for all animals. The evolution of the healing process was satisfactory. The variable presence and degree of erythema showed no statistically significant difference, although it was shown to be less in the control group on the days evaluated. The degree of erythema proved to be equivalent for the two anesthetics used, except on day 10, when the results were slightly higher in the lidocaine group.

The variable presence and degree of edema showed no statistically significant differences on days 3 and 10. In the same variable, however, some results showed statistically relevant differences on the first day of evaluation.

**Figure 2:**
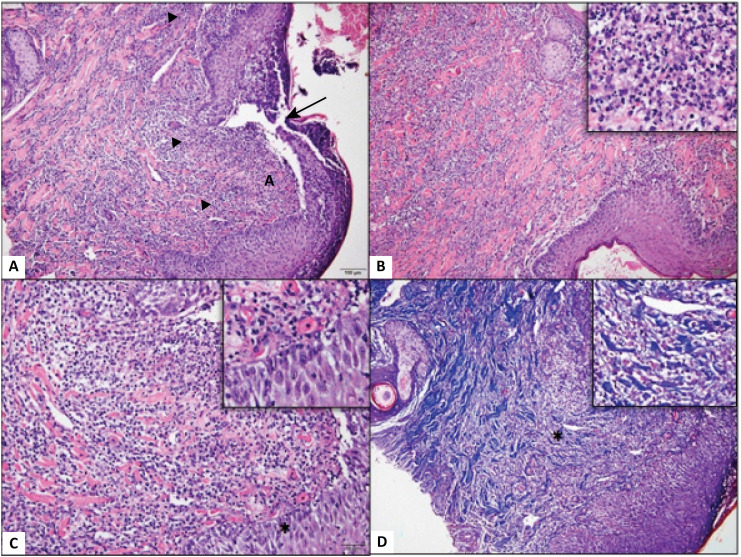
Photomicrograph of dog skin. (**A**) Skin with solution of continuity of the epidermis (*arrow*), associated with a marked neutrophilic inflammatory infiltrate (*arrowhead*) diffusely distributed through the dermis. (**B**) Note that the intense neutrophilic infiltrate (Detail, x20) is widely distributed in the superficial dermis. (**C**) Observe moderate edema of the epidermis evidenced by the increase in the space between the keratinocytes (*, Detail, x40), in addition to the marked inflammatory infiltrate. Hematoxylin and Eosin (x10). (**D**) Dissociation of collagen fibers from the dermis associated with intense and diffuse inflammatory infiltrate in the dermis (*, Detail, x20). Masson's trichrome, Obj. x10.

The microscopic variables of collagenization (p = 0.5542), neovascularization (p = 0.7541), edema (p = 0.7461), acute inflammatory cells and chronic inflammatory cells (p = 0.6908) did not obtain relevant statistical differences in the two experimental groups (Fig. 2).

In the group that was treated with lidocaine, two animals showed higher collagenization values in the control wound, while in three animals the values were higher in the treated lesion. In the group treated with bupivacaine, two animals demonstrated greater collagenization in the control group and five animals obtained better results in the treated group.

Neovascularization was observed on the tenth largest day in three animals in the control lesion in the lidocaine group and in three other animals in the treated lesion. In the bupivacaine group, the control lesion showed more pronounced neovascularization in five animals and in two animals in the treated lesion. All samples collected showed good vascularity, and in many of them the vessels were already mature. Thus, the lower values of neovascularization indicated vessels already fully formed, while higher values were used to indicate newly formed vessels.

The control lesion of four animals had more pronounced edema in the lidocaine group, and in six animals, the treated lesion was more edema. In seven animals, the control lesion had more edema in the bupivacaine group, in contrast to four animals that had the treated lesion more edema.

The degree of acute inflammatory cells was more pronounced in three animals in the control lesion and three in the lesion treated with lidocaine. In the bupivacaine group, five animals had a higher degree of acute inflammatory cells in the control group and only one in the treated lesion.

The lidocaine group showed only one animal with a higher degree of chronic inflammatory cells in the control lesion compared to six animals in the treated lesion. In the bupivacaine group, five animals showed a greater degree in the control lesion and four in the treated lesion.

## Discussion

According to Dogan *et al*.^13^, there is delay in wound healing with the use of lidocaine, and with the reduced tension strength of collagen fibers; other authors corroborate the macroscopic findings of this research, where no significant differences were observed between the healing processes^14^ ^,^ ^15^.

One of the main concerns in the topical use of anesthetics involves the cytotoxic effect on inflammatory cells and fibroblasts in vitro^16^ ^,^ ^17^. According to Bentov^18^, the inhibitory effect on the proliferation of fibroblasts occurred only in aged cells. The results obtained in this study show that there are no differences observed macroscopically during the healing period and there were also no significant differences at the end of the ten-day period, assessed microscopically on the histopathological examination. It is observed, however, that there were no elderly animals in this experiment, a factor that can be studied in the future in vivo.

The single application performed in the transsurgical period, mimics a common practice in the surgical routine and may have been a determining factor for the absence of side effects in healing, as studies show that the longer the duration of administration, the greater the damage to healing^18^. Additionally, considering our results, we can suggest that the concentration of the drug used may have been adequate in preventing side effects. The use of topical anesthetics is one of the techniques used to reduce intraoperative nociception or postoperative pain^5^ ^–^ ^7^. Its use in a single dose during surgery has the benefits of having low cost and absence of the need for injuries or additional procedures^19^ ^,^ ^20^. Therefore, the results found in this study, show that the concentration of the drug used may have been adequate to avoid side effects.

According to Busuioc *et al*.^20^ and Leong^21^, the time taken to perform the biopsy, ten days after surgery, mainly corresponds to the granulation-proliferation phase, a period that occurs when neovascularization occurs through the migration of endothelial cells and the migration, adhesion and proliferation of fibroblasts, which secrete collagen; this fact was proven in this study, since it was possible to visualize it ten days after the surgical procedure. Data by means of histology, presence of neovascularization, fibroblasts, as well as assessing collagenization were obtained.

Studies carried out on human wounds subjected to bupivacaine instillation show that there are no significant differences between treated and control lesions^22^. However, in veterinary medicine, these data are scarce. We did not find, nonetheless, in the present study, a significant difference between the groups regarding the use of drugs, the results being consistent with those described by Baxter^23^. On the other hand, less favorable interference of bupivacaine in relation to lidocaine was found in the variables edema, pruritus and neovascularization. In the lidocaine group, the performance was slightly lower in the variables erythema and degree of chronic inflammatory cells.

The results obtained in the present research indicate that the doses and concentrations of lidocaine and bupivacaine used do not have deleterious effects on skin healing. The use of local anesthetics in the surgical lesion, before suturing, is an important tool for pain control in the trans and postoperative period. The absence of adverse effects suggests that it is feasible the intraoperative use of local anesthetics as an aid in the control of cutaneous pain.

## Conclusion

The topical application of lidocaine and bupivacaine in the surgical wound is feasible and it does not influence skin healing. The benefit of such a practice, which has been the subject of other studies, seems to outweigh the risks.
